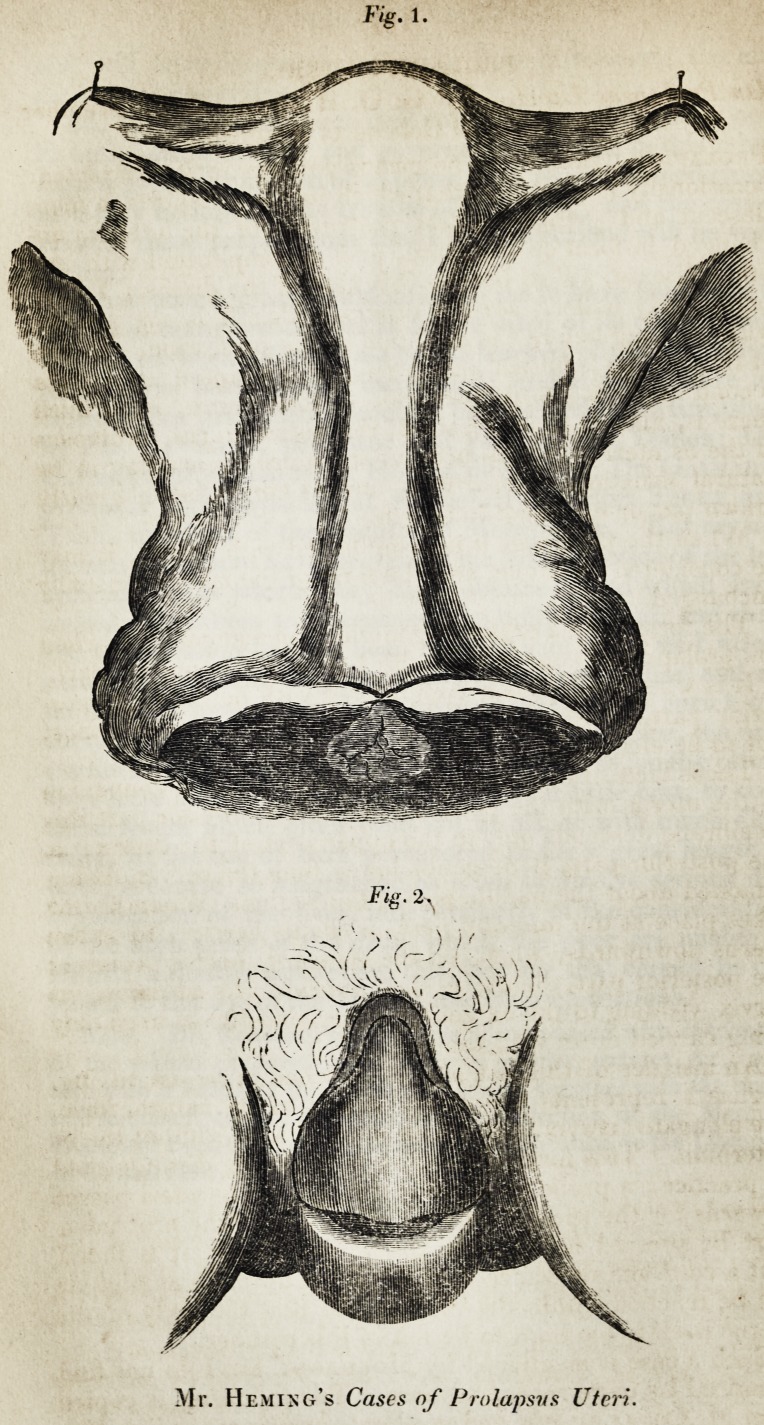# On Prolapsus Uteri

**Published:** 1832-08

**Authors:** G. O. Heming

**Affiliations:** Member of the Royal College of Surgeons.


*Fig.* 1.

*Fig.* 2.

Mr. Heming's *Cases of Prolapsus Uteri.*

*On Prolapsus Uteri.* By G. O. Heming, Esq., Member of the Royal College of Surgeons.

Prolapsus uteri is by no means uniform in its character: occasionally it consists in that which its name imports, a mere descent and greater or less protrusion of the uterus, unchanged in form or structure, and uncomplicated with any other disease. But, besides this kind of the disease, I have particularly noticed several others, which it is my present wish to bring before the attention of the profession. In one of these there is hernia anterior to the prolapsed uteri; in a second there is hernia posterior to that organ; in a third there is, instead of prolapsus of the entire uterus, prolapsus of the os uteri only, the body of the uterus remaining in its natural situation, or nearly so, and its cervix being greatly drawn out or extended in length.

When the prolapsus is uncomplicated with hernia, it may be considered as simple, and the form of the uterus is usually unchanged.

The form of this organ is generally unchanged also when the prolapsus is complicated with hernia at its anterior part.

It is when prolapsus is complicated with hernia at its posterior part, I believe, that the cervix of the uterus is most apt to be elongated.

These facts appear to depend upon the natural connexion of the uterus with the peritoneum: in the anterior part this membrane descends only to the upper part of the cervix; at the posterior part, it descends lower than the cervix, being attached about the middle part of the vagina. When hernia takes place at the anterior part, it simply carries the entire uterus downwards; when, on the contrary, hernia occurs at the posterior part, the uterus may remain in its situation, its cervix yielding to the extending force, and the os uteri only being carried down with the hernia.

An instance of this last case is sketched in the engraving. Figure 1 represents the uterus nearly of its natural form, the elongated cervix, and the os uteri as seen without the os externum. This form of the disease is readily distinguished in practice: a probe introduced through the os uteri passes upwards for the space of five or six inches; if the protruded part be pressed between the thumb and finger, it is found that a cord-like substance may be traced upwards as high as can be reached within the vagina, and that the body of the uterus itself is too high to be felt in this manner.

Such a case is mentioned by Morgagni, but I do not find it noticed by more recent authors. The following is copied from Mr. Cooke's useful and excellent abridgment, v. ii. p. 493.

"An aged woman, at Bologna, had been afflicted with hemiplegia, in consequence of which, for many years, she had been unable to move one side of her body; and at length the power of motion in the other side was likewise abolished; a round body also protruded from the vagina. Ultimately she was seized with inflammation of the thorax, and died at this hospital in 1704.

"*Dissection.* The head exhibited nothing worthy of notice, except an accumulation of serum between the dura and pia mater. The thorax was not examined. I observed that the fundus uteri occupied a lower situation within the pelvis than usual, but its descent was not such as to lead me to suppose the mouth of the uterus so low as it actually was. The labia pudendi were greatly dilated, and a body, three or four digits in length, protruded. This substance was of a cylindrical form, very thick, and its texture resembled that of ligament, except at the bottom, where ulceration had taken place. I perceived that it was the vagina inverted. At the upper and anterior part of this prolapsed body, was the orifice of the urethra; and beneath this aperture, on each side, the considerably dilated mouth of a lacuna was visible. In the middle of the lower part there was an orifice, through which the os uteri could be distinguished at no great distance; and I passed a probe through it without any great difficulty to the upper parietes of the cavity of the uterus. Surprised at the unusual length of this organ, I cut into the vagina, and within it lay the cervix uteri, extremely lengthened. We could not wonder at this elongation, because the parietes of the cervix itself, as well as those of the fundus, instead of being firm, as they naturally are, were extremely relaxed and flabby; and all the other parts belonging to the uterus, and situated within the pelvis, were in the same condition." (*Morgagni,* xlv. ii.)

Figure 2 represents a case of prolapsed uterus complicated with hernia of the bladder anteriorly, and with a tumor consisting of a pouch of the rectum, filled with feculent matter, posteriorly. It is the case in which I operated in the manner suggested by Dr. Marshall Hall: the case is given by that gentleman in the Medical Gazette for November 26, 1831, which I must transcribe in this place.

"The subject of the case which I am about to detail, was a poor woman, whose bread depended upon the labour of her hands. Her sufferings, from the prolapsed state of the uterus, were often extreme, and she was frequently disabled from engaging in her various occupations.

"For several years there had been complete prolapsus of the uterus; to this were also conjoined a partial descent of the bladder at the anterior, and of the rectum, formed into a pouch, at the posterior part of this prolapsus. The os uteri protruded at least two inches beyond the os externum.

"It occurred to me that, if the canal of the vagina could be considerably, permanently, and firmly, reduced in its diameter, the uterus would be supported in its place, and prevented from resuming its prolapsed situation; and that this might be done by removing a portion of its mucous membrane along the anterior part, and by bringing and returning the denuded surfaces in contact by successive deep sutures, until they should unite by cicatrix.

"This operation was performed by Mr. Heming, of Kentish Town. The uterus being protruded as much as possible, by the efforts of the patient, two parallel incisions were made through the mucous membrane, from the sides of the os uteri, along the course of the protruded vagina to the os externum, the portion of this membrane situated between these incisions was then removed, leaving a space of one inch and a half in breadth, and of the entire length of the vagina, completely denuded. A suture was then inserted, near the os uteri. This suture being tightened, the os uteri was obviously pushed upwards. A second, a third, and other ligatures, were then inserted, in the same manner, at short intervals, to the os externum; each ligature, on being tightened, moving and supporting the os uteri upwards.

"This operation was attended with little pain; the only sensitive parts of the membrane being those near the os uteri and os externum.

"The patient was directed to keep quiet in bed. The bowels had been opened. An opiate was given. No pain or fever followed. In four or five weeks the denuded parts had firmly united, and shortly afterwards the ligatures were come away.

"On examination, six, eight, and ten weeks after the operation, the os uteri could be just felt in situ, by the finger passed through the vagina: the vagina was firmly contracted along its whole course.

"The prolapsus of the uterus was thus completely remedied. The descent of the pouch of the rectum was lessened.

"P.S. The principle upon which this case was treated is illustrated by a fact, detailed to me by Dr. Holland, of Queen street, Mayfair. A pessary introduced in a young person to support the uterus, subject to be completely prolapsed, induced great inflammation. This was followed by such firm contraction of the vagina, that the uterus ever afterwards remained in its proper situation."

I have this day examined this poor woman. The vagina retains its dimensions; the uterus is kept in its place; the bladder is also supported in its proper situation; the pouch of the rectum is the only part of the malady unremedied.

It appears, therefore, that prolapsus uteri may occur under the following forms: that of, 1. Simple prolapsus uteri. 2. Prolapsus with hernia at its anterior part. 3. Prolapsus with hernia at the posterior part. 4. Prolapsus oris uteri.

The two former cases, and the two last cases, are apt to occur together respectively. It is to the first cases that the operation which has been described seems to be best adapted; it remains to be ascertained how far it may be equally successful in the others.

## Figures and Tables

**Fig. 1. Fig. 2. f1:**